# Clinical outcomes during and beyond different COVID-19 critical illness variant periods compared with other lower respiratory tract infections

**DOI:** 10.1186/s13054-023-04722-0

**Published:** 2023-11-06

**Authors:** Pontus Hedberg, Nicholas Baltzer, Fredrik Granath, Michael Fored, Johan Mårtensson, Pontus Nauclér

**Affiliations:** 1 Department of Medicine, Huddinge, Karolinska Institutet, H7 Medicin, Huddinge, H7 Infektion och Hud Sönnerborg, 171 77 Stockholm, Sweden; 2 Division of Infectious Diseases, Department of Medicine, Solna, Karolinska Institutet, Stockholm, Sweden; 3 Department of Medical Epidemiology and Biostatistics, Karolinska Institutet, Stockholm, Sweden; 4 Clinical Epidemiology Division, Department of Medicine Solna, Karolinska Institutet, Stockholm, Sweden; 5 Department of Physiology and Pharmacology, Karolinska Institutet, Stockholm, Sweden; 6 Department of Perioperative Medicine and Intensive Care, Karolinska University Hospital, Stockholm, Sweden; 7 Department of Infectious Diseases, Karolinska University Hospital, Stockholm, Sweden

**Keywords:** COVID-19, SARS-CoV-2, Intensive care, Critical illness, Long-term outcomes

## Abstract

**Background:**

It is yet to be better understood how outcomes during and after the critical illness potentially differ between severe acute respiratory syndrome coronavirus 2 (SARS-CoV-2) variants from other lower respiratory tract infections (LRTIs). We aimed to compare outcomes in adults admitted to an intensive care unit (ICU) with coronavirus disease 2019 (COVID-19) during the Wild-type, Alpha, Delta, and Omicron periods with individuals admitted with other LRTI.

**Methods:**

Population-based cohort study in Stockholm, Sweden, using health registries with high coverage, including ICU-admitted adults from 1 January 2016 to 15 September 2022. Outcomes were in-hospital mortality, 180-day post-discharge mortality, 180-day hospital readmission, 180-day days alive and at home (DAAH), and incident diagnoses registered during follow-up.

**Results:**

The number of ICU admitted individuals were 1421 Wild-type, 551 Alpha, 190 Delta, 223 Omicron, and 2380 LRTI. In-hospital mortality ranged from 28% (n = 665) in the LRTI cohort to 35% (n = 77) in the Delta cohort. The adjusted cause-specific hazard ratio (CSHR) compared with the LRTI cohort was 1.33 (95% confidence interval [CI] 1.16–1.53) in the Wild-type cohort, 1.53 (1.28–1.82) in the Alpha cohort, 1.70 (1.30–2.24) in the Delta cohort, and 1.59 (1.24–2.02) in the Omicron cohort. Among patients discharged alive from their COVID-19 hospitalization, the post-discharge mortality rates were lower (1–3%) compared with the LRTI cohort (9%), and the risk of hospital readmission was lower (CSHRs ranging from 0.42 to 0.68). Moreover, all COVID-19 cohorts had compared with the LRTI cohort more DAAH after compared with before the critical illness.

**Conclusion:**

Overall, COVID-19 critical was associated with an increased hazard of in-hospital mortality, but among those discharged alive from the hospital, less severe long-term outcomes were observed compared with other LRTIs.

**Supplementary Information:**

The online version contains supplementary material available at 10.1186/s13054-023-04722-0.

## Introduction

Mortality during coronavirus disease 2019 (COVID-19) critical illness is substantial, with mortality rates approaching or exceeding 30% [1–4]. Since the ancestral strain of severe acute respiratory syndrome coronavirus 2 (SARS-CoV-2), five variants of concerns have been identified: Alpha, Beta, Gamma, Delta, and Omicron [5]. Whilst an extensive body of evidence have demonstrated acute infections with Omicron to be less severe compared with preceding SARS-CoV-2 variants [6], similar mortality rates for intensive care unit (ICU) admitted patients have been reported regardless of variant [7, 8]. Two French studies including patients admitted to ICUs for severe COVID-19 observed no difference in in-ICU mortality or 28-day mortality between the Delta and the Omicron period [7, 8]. However, a Brazilian study including ICU-admitted COVID-19 patients observed a lower 60-day mortality during a period of Omicron dominance compared with periods of nonvariant dominance and Gamma and Delta dominance, respectively [9]. Irrespective of SARS-CoV-2 variant and a substantial critical illness mortality, most patients receiving intensive care treatment for COVID-19 survive their acute illness episode.

It is yet to be better understood how outcomes during and after the critical illness potentially differ between SARS-CoV-2 variants from other lower respiratory tract infections (LRTIs). This is important to identify potentially different recovery trajectories and to facilitate planning of follow-up strategies for patients having experienced COVID-19 critical illness compared with other LRTIs [10]. The SARS-CoV-2 virus has developed substantially throughout the pandemic, motivating characterizations of outcomes of COVID-19 critical illness during different variant periods. These types of investigations are often hampered by lack of population-based data on mortality, inpatient care, outpatient specialist care, and primary care. The aim of this study was to use population-based health registries to compare outcomes during and beyond COVID-19 critical illness overall as well as for different SARS-CoV-2 variant periods compared with other LRTIs.

## Methods

### Study design, setting, and population

Stockholm County in Sweden has a population of 2.4 million inhabitants, served by six acute care hospitals with ICUs. Throughout the pandemic, every hospital in Stockholm County expanded its ICUs, ensuring ICU capacity was never surpassed. It is crucial to note that the criteria for ICU admission remained consistent for both COVID-19 and non-COVID-19 patients. However, as highlighted in a prior study by Strålin et al., the percentage of hospitalized patients admitted to the ICU diminished over time [11]. This suggests a possibility that, as time progressed, the hospitalized COVID-19 population exhibited milder symptoms. Furthermore, as treatment strategies for COVID-19 evolved, intermediate care wards began accommodating patients with more severe conditions.

We conducted a population-based retrospective cohort study including adults admitted to these ICUs with COVID-19 or other LRTIs. We identified all individuals aged 18 years or older who were hospitalized and treated in the ICU any time from 1 January 2016 to 15 September 2022. Only individuals who had lived in Stockholm County at least 1 year before the hospital admission were considered to enable classification of underlying health status. We identified all hospitalizations where an International Statistical Classification of Diseases and Related Health Problems 10^th^ Revision (ICD-10) code of COVID-19 (U07.1 or U07.2) or other LRTIs (influenza [J09.X-J11.X] and other LRTIs [J12.X- J18.X]) had been registered during ICU treatment. For the COVID-19 cohort, a polymerase chain reaction (PCR) test positive for SARS-CoV-2 any time from 14 days before hospital admission or during the hospitalization was required. Patients with a LRTI ICD-10 diagnosis and a positive SARS-CoV-2 test were excluded from the analyses. Only the first hospitalization per individual and cohort (COVID-19 or LRTI) were included. All hospitalizations meeting these criteria were included in the analyses of in-hospital mortality. Individuals who then were discharged alive from the hospital any time up until 18 April 2022 were included in the analyses of long-term outcomes. This date was selected to allow for 180 days of follow-up before administrative censoring (15 October 2022).

### Cohorts

Six cohorts were used in the study: All COVID-19, Wild-type, Alpha, Delta, Omicron, and LRTI. The SARS-CoV-2 variant cohorts (Wild-type, Alpha, Delta, Omicron), were defined based on the date of the PCR-positive test, using the same variant periods as previously described by the Public Health Agency of Sweden (see Additional file 1: Table S1) [12]. If an individual had a sample sequenced for any of these variants outside of the corresponding variant period, the variant classification was based on the sequenced sample.

### Data sources

Data were linked using personal identification numbers, unique for each Swedish resident, from the Swedish Intensive Care Registry (SIR), the Stockholm regional healthcare data warehouse (VAL), the Registry of notifiable disease (SmiNet), the National Vaccination Register (NVR), and Statistics Sweden. SIR is an intensive care quality registry recording all ICU admissions, including data on diagnoses, medical procedures, and measurements of disease severity [3]. VAL contains data from healthcare databases within the Stockholm Region, including demographics, migration, drug prescriptions, and data on all inpatient stays and outpatient visits reimbursed by Region Stockholm [13]. This includes near complete coverage of specialist care and 94% of primary care [13]. SmiNet contains all PCR SARS-CoV-2 positive test results notified in accordance with the Communicable Diseases Act [14]. The data from NVR included all COVID-19 vaccinations administered in Sweden to the Stockholm County population. Data from Statistics Sweden were used to collect sociodemographic data [15]. The data sources are described in more detail in Text S1.

### Outcomes and variable definitions

In-hospital all-cause mortality was analysed up until discharge from hospital. Long-term outcomes were analysed from the time of discharge alive and the following 180-days. The long-term outcomes were all-cause mortality, all-cause hospital readmission, days alive and at home (DAAH) [16], and the ten most common incident diagnoses registered for each cohort within all healthcare during the follow-up period of 180 days. DAAH was defined as the number of days not admitted to hospital, not attending an outpatient care facility, not living in a nursing home or receiving home care services, and not calling the Swedish National Medical Advisory Service (called 1177 in Swedish). Incident diagnoses were defined as diagnoses registered by a physician which the individual did not have registered during a period of 3 years before or during the critical illness hospital admission. Finally, to get a better understanding of the overall mortality, irrespective if in-hospital or out-of-hospital, we analyzed the 180-day all-cause mortality from the day of admission. Similar to the cut-offs used for the other long-term outcomes, individuals who were admitted to the ICU any time up until 18 April 2022 were included to allow for 180 days of follow-up before administrative censoring (15 October 2022). Individuals were followed from the day of ICU admission to the day of death, moving out of the region, or 180 days, whichever occurred first.

Comorbidities were based on conditions with increased risk of severe COVID-19 [17]. Data on disposable income quartile were based on information from the year before the hospital admission (or from 2019 for individuals admitted in 2021 or 2022), with quartiles being calculated separately for each birthyear in the entire Stockholm County population. COVID-19 vaccination status was based on the number of doses received until fourteen days before the hospital admission. Classification of procedures in the ICU were based on national procedure codes (Swedish: KVÅ codes). All study outcomes and other collected variables are further described in Additional file 1: Table S1.

### Statistical methods

We described characteristics of the six cohorts: All COVID-19, SARS-CoV-2 Wild-type, Alpha, Delta, or Omicron variants, and LRTI. Continuous values were presented as medians with interquartile range (IQR), and categorical values as numbers with percentages.

The crude 120-day cumulative incidences of in-hospital mortality and alive hospital discharge were estimated with the Aalen and Johansen estimator for each cohort [18]. These results were presented overall as well as stratified on age (18–64 years and ≥ 65 years). Multivariable cause-specific Cox-proportional hazards regression models as well as Fine-Gray subdistribution hazard models were used to compare the in-hospital mortality in the All COVID-19, Wild-type, Alpha, Delta, and Omicron cohorts with the LRTI cohort [19, 20]. Models were adjusted for age, sex, region of birth, disposable income quartile, and comorbidities (cancer, cardiac or cerebrovascular disease, chronic kidney failure, chronic liver disease, chronic lung disease, diabetes, hypertension, immunocompromised state, mental health disorder, neurological disease, and obesity). Age was included as a continuous variable using restricted cubic splines with four knots [21]. Cause-specific hazard ratios (CSHRs) and subdistribution hazard ratios (SHR) with 95% confidence intervals (CIs) were presented. To further investigate the mortality across different age strata, age-stratified crude mortality rates were also presented.

Characteristics of individuals discharged alive from the hospital up until 18 April 2022 in the six cohorts were then described. For these individuals, comorbidity classification was based on diagnosis codes until the day of hospital discharge, not until the day before hospital admission as in the cohorts for in-hospital mortality. Individuals were censored at 180 days from the day after hospital discharge, moving out of the region or death, whichever occurred first. Multivariable Cox-proportional hazards regression models were used to investigate 180-day post-discharge mortality and 180-day hospital readmission in the COVID-19 cohorts compared with the LRTI cohort. Furthermore, Fine-Gray subdistribution hazard models were also used for hospital readmission to further investigate the competing risk of death. For 180-day post-discharge mortality, adjustments were only made for age and sex due to the low number of events observed, whereas age, sex, region of birth, disposable income quartile, and comorbidities were used for hospital readmission. The ten most common diagnoses for the first hospital readmission were also presented for each cohort. DAAH 180 days after compared with 180 days before the critical illness episode was analysed descriptively (medians and IQRs) as well as modelled with adjusted difference-in-differences analyses, a modelling strategy based on linear probability models [22]. These models were also adjusted for age, sex, region of birth, disposable income quartile, and comorbidities. Results from the models were presented with 95% CIs based on robust standard errors.

The cumulative incidences of 180-day mortality from the day of ICU-admission (irrespective if in-hospital or out-of-hospital) were also described overall and age-stratified (18–64 years and ≥ 65 years). as for in-hospital mortality.

Low levels of missing data were observed for region of birth (< 0.1% missing) and disposable income (0.2% missing). This was classified as missing data and all individuals were included in all models. Among variables used for descriptive purposes only, data were missing for time to ICU admission from COVID-19 symptom onset (2%), partial pressure of oxygen divided by fraction of inspired oxygen (PaO_2_/FiO_2_) (16%), and hospital length of stay (individuals not discharged by 15 October 2022) (0.2%).

Statistical analyses were conducted using R version 4.1.0.

## Results

### Study population

**Fig. 1:**
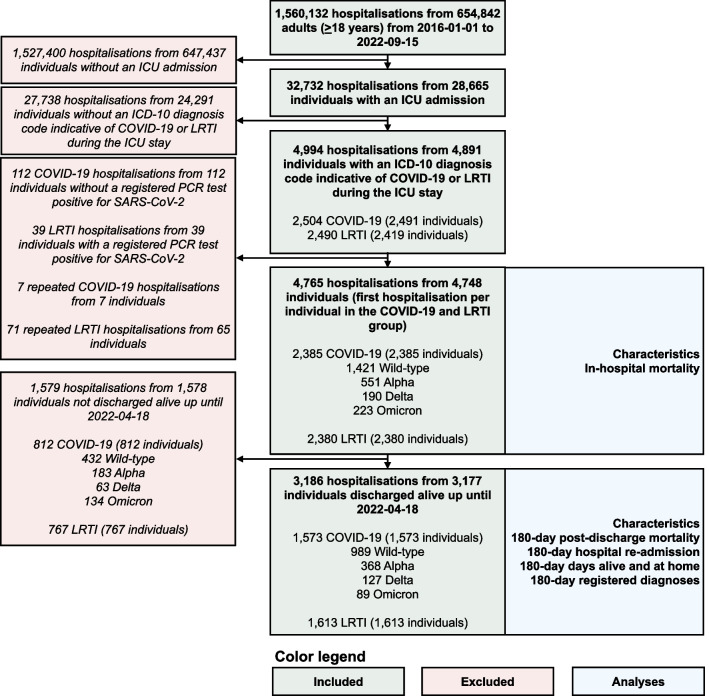
Study flow chart. *Note* The green boxes indicate the included hospitalizations and the red boxed indicate the excluded hospitalizations. The blue boxes describe what analyses were performed for each part of the study population. *COVID-19* Coronavirus disease 2019, *ICD-10* International Statistical Classification of Diseases and Related Health Problems 10th Revision, *ICU* Intensive care unit, *LRTI* Lower respiratory tract infection, *PCR* Polymerase chain reaction, *SARS-CoV-2* Severe acute respiratory syndrome coronavirus 2

A total of 4765 hospitalizations from 4748 individuals were included: 1421 Wild-type, 551 Alpha, 190 Delta, 223 Omicron, and 2380 LRTI (Fig. 1). The number of ICU-admitted hospitalizations per calendar month is presented for each cohort in Additional file 1: Fig. S1. The median (IQR) age was 63 (54–72) in the All COVID-19 cohort, 63 (54–71) years in the Wild-type cohort, 64 (54–72) years in the Alpha cohort, 62 (49–71) years in the Delta cohort, 68 (54–77) years in the Omicron cohort, and 67 (56–75) years in the LRTI cohort (Table 1). Of the SARS-CoV-2 variants, patients with Omicron were most similar to the LRTI cohort, with more than 50% being aged 65 years or older and around 20% of patients being immunocompromised. A total of 74% (n = 140) and 31% (n = 70) of individuals in the Delta and Omicron cohorts were unvaccinated before the hospital admission, respectively. Characteristics of unvaccinated (or one dose) Delta and Omicron patients compared with patients having received at least two doses are shown in Additional file 1: Table S2. A total of 36% (n = 79) of the unvaccinated patients were ≥ 65 years, compared with 62% (n = 118) of patients having received ≥ 2 doses. Of the 2380 LRTI hospitalizations, 246 were caused by influenza and 2134 by other LRTIs. Characteristics of these groups are presented in Additional file 1: Table S3. The median (IQR) age was 64 (51–74) for the influenza group and 67 (56–75) for the other LRTIs group. The comorbidity burden as well as the ICU characteristics were rather similar for the two groups.

**Table 1:** Characteristics of the cohorts

Variable	All COVID-19 (n = 2385)	Wild-type (n = 1421)	Alpha (n = 551)	Delta (n = 190)	Omicron (n = 223)	LRTI (n = 2380)
*Baseline characteristics*						
Male sex	1684 (70.6)	1043 (73.4)	367 (66.6)	136 (71.6)	138 (61.9)	1506 (63.3)
Age, years	63.0 [54.0, 72.0]	63.0 [54.0, 71.0]	64.0 [54.0, 72.0]	61.5 [49.2, 71.0]	68.0 [54.0, 76.5]	67.0 [56.0, 75.0]
18–44	267 (11.2)	133 (9.4)	68 (12.3)	31 (16.3)	35 (15.7)	300 (12.6)
45–64	1030 (43.2)	650 (45.7)	230 (41.7)	86 (45.3)	64 (28.7)	734 (30.8)
65 or older	1088 (45.6)	638 (44.9)	253 (45.9)	73 (38.4)	124 (55.6)	1346 (56.6)
Region of birth						
Africa	171 (7.2)	114 (8.0)	31 (5.6)	16 (8.4)	10 (4.5)	71 (3.0)
The Americas	124 (5.2)	85 (6.0)	27 (4.9)	7 (3.7)	5 (2.2)	27 (1.1)
Asia or Oceania	547 (22.9)	366 (25.8)	100 (18.1)	53 (27.9)	28 (12.6)	178 (7.5)
Europe	368 (15.4)	210 (14.8)	87 (15.8)	39 (20.5)	32 (14.3)	302 (12.7)
Sweden	1172 (49.1)	646 (45.5)	305 (55.4)	75 (39.5)	146 (65.5)	1801 (75.7)
Missing	3 (0.1)	0 (0.0)	1 (0.2)	0 (0.0)	2 (0.9)	1 (0.0)
Yearly disposable income quartile						
Quartile 1	920 (38.6)	520 (36.6)	201 (36.5)	94 (49.5)	105 (47.1)	1007 (42.3)
Quartile 2	626 (26.2)	380 (26.7)	145 (26.3)	48 (25.3)	53 (23.8)	588 (24.7)
Quartile 3	479 (20.1)	298 (21.0)	111 (20.1)	29 (15.3)	41 (18.4)	423 (17.8)
Quartile 4	353 (14.8)	223 (15.7)	93 (16.9)	17 (8.9)	20 (9.0)	360 (15.1)
Missing	7 (0.3)	0 (0.0)	1 (0.2)	2 (1.1)	4 (1.8)	2 (0.1)
Cancer	127 (5.3)	72 (5.1)	27 (4.9)	8 (4.2)	20 (9.0)	327 (13.7)
Cardiac or cerebrovascular disease	503 (21.1)	275 (19.4)	115 (20.9)	35 (18.4)	78 (35.0)	770 (32.4)
Chronic kidney failure	174 (7.3)	86 (6.1)	36 (6.5)	16 (8.4)	36 (16.1)	212 (8.9)
Chronic liver disease	67 (2.8)	39 (2.7)	16 (2.9)	3 (1.6)	9 (4.0)	160 (6.7)
Chronic lung disease	182 (7.6)	100 (7.0)	44 (8.0)	12 (6.3)	26 (11.7)	451 (18.9)
Diabetes (type 1 or 2)	626 (26.2)	380 (26.7)	135 (24.5)	50 (26.3)	61 (27.4)	540 (22.7)
Hypertension	1057 (44.3)	638 (44.9)	240 (43.6)	73 (38.4)	106 (47.5)	1115 (46.8)
Immunocompromised state	288 (12.1)	153 (10.8)	61 (11.1)	27 (14.2)	47 (21.1)	437 (18.4)
Mental health disorder	71 (3.0)	36 (2.5)	16 (2.9)	4 (2.1)	15 (6.7)	138 (5.8)
Neurological disease	60 (2.5)	34 (2.4)	7 (1.3)	4 (2.1)	15 (6.7)	176 (7.4)
Obesity	362 (15.2)	187 (13.2)	120 (21.8)	27 (14.2)	28 (12.6)	183 (7.7)
Charlson comorbidity index score	0.0 [0.0, 2.0]	0.0 [0.0, 2.0]	0.0 [0.0, 2.0]	0.0 [0.0, 1.0]	1.0 [0.0, 3.0]	1.0 [0.0, 3.0]
0	1265 (53.0)	761 (53.6)	293 (53.2)	120 (63.2)	91 (40.8)	868 (36.5)
1–2	732 (30.7)	450 (31.7)	172 (31.2)	43 (22.6)	67 (30.0)	708 (29.7)
3–4	227 (9.5)	124 (8.7)	48 (8.7)	17 (8.9)	38 (17.0)	432 (18.2)
≥ 5	161 (6.8)	86 (6.1)	38 (6.9)	10 (5.3)	27 (12.1)	372 (15.6)
COVID-19 vaccination status before hospitalization						
Unvaccinated	2158 (90.5)	1421 (100.0)	527 (95.6)	140 (73.7)	70 (31.4)	NA
One dose	31 (1.3)	0 (0.0)	20 (3.6)	5 (2.6)	6 (2.7)	NA
Two doses	106 (4.4)	0 (0.0)	4 (0.7)	41 (21.6)	61 (27.4)	NA
Three doses	67 (2.8)	0 (0.0)	0 (0.0)	4 (2.1)	63 (28.3)	NA
Four doses	23 (1.0)	0 (0.0)	0 (0.0)	0 (0.0)	23 (10.3)	NA
*ICU characteristics*						
Time to ICU admission from COVID-19 symptom onset	10.0 [7.0, 13.0]	10.0 [7.0, 13.0]	10.0 [7.0, 13.0]	10.0 [7.0, 14.0]	3.0 [0.0, 8.0]	NA
Missing	49 (2.1)	18 (1.3)	16 (2.9)	2 (1.1)	13 (5.8)	NA
SAPS 3 score	56.0 [49.0, 65.0]	56.0 [48.0, 63.0]	57.0 [51.0, 66.0]	56.0 [49.2, 66.0]	59.0 [51.0, 70.5]	63.0 [54.0, 73.0]
PaO_2_/FiO_2_ ratio	11.4 [8.9, 15.6]	11.4 [9.0, 15.3]	10.6 [8.5, 13.8]	11.2 [8.6, 15.0]	18.2 [11.2, 38.0]	19.3 [12.1, 30.3]
Missing	292 (12.2)	179 (12.6)	50 (9.1)	17 (8.9)	46 (20.6)	498 (20.9)
Mechanical ventilation	1379 (57.8)	878 (61.8)	287 (52.1)	105 (55.3)	109 (48.9)	1307 (54.9)
Duration	10.9 [5.0, 21.2]	11.9 [6.1, 22.3]	11.2 [5.1, 22.5]	9.3 [4.8, 14.8]	3.6 [0.7, 9.5]	7.2 [3.0, 15.3]
NIV or HFNO	1616 (67.8)	903 (63.5)	451 (81.9)	155 (81.6)	107 (48.0)	1332 (56.0)
Prone positioning	989 (41.5)	651 (45.8)	225 (40.8)	85 (44.7)	28 (12.6)	104 (4.4)
Length of stay in ICU	7.9 [2.9, 17.2]	9.0 [3.7, 19.0]	6.6 [2.9, 16.5]	8.1 [3.2, 16.3]	2.7 [0.9, 7.5]	4.7 [1.7, 12.8]
Length of stay in hospital	21.0 [12.0, 39.0]	22.0 [13.0, 41.0]	21.0 [12.0, 40.0]	19.0 [13.0, 32.0]	14.0 [7.0, 29.0]	19.0 [9.0, 39.0]
Missing	3 (0.1)	1 (0.1)	0 (0.0)	0 (0.0)	2 (0.9)	7 (0.3)

Numeric values are presented as median [interquartile range], and categorical values are presented as number (percentage)

*COVID-19* Coronavirus disease 2019, *FiO*_*2*_ Fraction of inspired oxygen, *HFNO* High flow nasal oxygen, *ICU* Intensive care unit, *LRTI* Lower respiratory tract infection, *NA* Not applicable, *NIV* Non-invasive ventilation, *PaO*_*2*_ Partial pressure of oxygen, *SAPS 3* Simplified acute physiology score 3

### In-hospital mortality

**Fig. 2:**
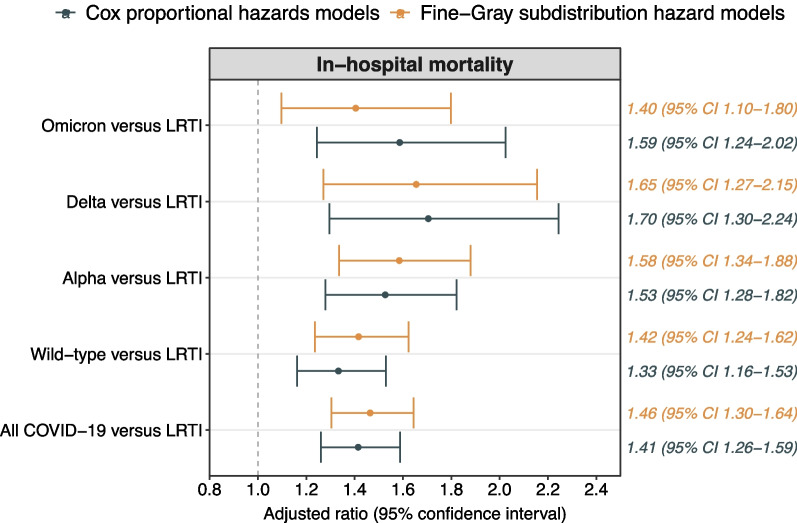
Alive hospital discharge and in-hospital all-cause mortality in the cohorts. Adjusted cause-specific hazard ratios (95% confidence interval) and subdistribution hazard ratios for in-hospital all-cause mortality in the All COVID-19, Wild-type, Alpha, Delta, and Omicron cohort compared with the LRTI cohort. The model was adjusted for age, sex, region of birth, yearly disposable income quartile, and all studied comorbidities (cancer, cardiac or cerebrovascular disease, chronic kidney failure, chronic liver disease, chronic lung disease, diabetes, hypertension, immunocompromised state, mental health disorder, neurological disease, and obesity). *CI* Confidence interval, *COVID-19* Coronavirus disease 2019, *LRTI* Lower respiratory tract infection

In-hospital mortality was 32% (n = 754) in the All COVID-19 cohort, 30% (n = 431) in the Wild-type cohort, 33% (n = 183) in the Alpha cohort, 33% (n = 63) in the Delta cohort, 35% (n = 77) in the Omicron cohort, and 28% (n = 665) in the LRTI cohort. When compared with the LRTI cohort, the adjusted CSHR was 1.42 (1.27–1.59) in the All COVID-19 cohort, 1.34 (1.17–1.54) in the Wild-type cohort, 1.54 (1.29–1.84) in the Alpha cohort, 1.72 (1.31–2.26) in the Delta cohort, and 1.59 (1.24–2.03) in the Omicron cohort (Fig. 2). The adjusted SHRs were similar to the adjusted CSHRs. Age-stratified in-hospital mortality rates are presented in Additional file 1: Figs. S2 and S3. Differences in mortality between COVID-19 and LRTI was more pronounced among individuals aged 65 years or older. The in-hospital mortality was 22% (n = 55) in the influenza group and 29% (n = 610) in the other LRTIs group.

### Long-term outcomes

A total of 989 Wild-type, 368 Alpha, 127 Delta, 89 Omicron, and 1613 LRTI episodes were included in the follow-up after discharge from hospital (Table 2). The median (IQR) age ranged from 56 (47–67) years for patients with Delta to 64 (52–73) years for LRTI. The median (IQR) length of stay in the ICU was 7 (3–16) days in the All COVID-19 cohort, with the shortest stay for Omicron [2 (1–5) days], and 4 (2–12) days in the LRTI cohort. Of the 1613 LRTI hospitalizations, 189 were caused by influenza and 1424 by other LRTIs. A total of 40% (n = 76) of were aged 65 years or older in the influenza group, compared with 50% (n = 718) in the other LRTIs group (Additional file 1: Table S4).

**Table 2:** Characteristics of the cohorts discharged alive from the hospital

Variable	All COVID-19 (n = 1573)	Wild-type (n = 989)	Alpha (n = 368)	Delta (n = 127)	Omicron (n = 89)	LRTI (n = 1613)
*Baseline characteristics*						
Male sex	1076 (68.4)	703 (71.1)	237 (64.4)	92 (72.4)	44 (49.4)	1008 (62.5)
Age, years	60.0 [50.0, 68.0]	60.0 [52.0, 67.0]	60.5 [48.0, 68.0]	56.0 [46.5, 66.5]	61.0 [47.0, 74.0]	64.0 [52.0, 73.0]
18–44	233 (14.8)	122 (12.3)	65 (17.7)	28 (22.0)	18 (20.2)	263 (16.3)
45–64	808 (51.4)	534 (54.0)	176 (47.8)	63 (49.6)	35 (39.3)	556 (34.5)
65 or older	532 (33.8)	333 (33.7)	127 (34.5)	36 (28.3)	36 (40.4)	794 (49.2)
Region of birth						
Africa	123 (7.8)	84 (8.5)	21 (5.7)	12 (9.4)	6 (6.7)	53 (3.3)
The Americas	86 (5.5)	61 (6.2)	18 (4.9)	4 (3.1)	3 (3.4)	16 (1.0)
Asia or Oceania	379 (24.1)	253 (25.6)	73 (19.8)	38 (29.9)	15 (16.9)	138 (8.6)
Europe	233 (14.8)	142 (14.4)	52 (14.1)	24 (18.9)	15 (16.9)	203 (12.6)
Sweden	750 (47.7)	449 (45.4)	204 (55.4)	49 (38.6)	48 (53.9)	1203 (74.6)
Missing	2 (0.1)	0 (0.0)	0 (0.0)	0 (0.0)	2 (2.2)	0 (0.0)
Yearly disposable income quartile						
Quartile 1	595 (37.8)	355 (35.9)	129 (35.1)	61 (48.0)	50 (56.2)	712 (44.1)
Quartile 2	419 (26.6)	272 (27.5)	101 (27.4)	31 (24.4)	15 (16.9)	393 (24.4)
Quartile 3	317 (20.2)	209 (21.1)	75 (20.4)	21 (16.5)	12 (13.5)	280 (17.4)
Quartile 4	236 (15.0)	153 (15.5)	62 (16.8)	12 (9.4)	9 (10.1)	228 (14.1)
Missing	6 (0.4)	0 (0.0)	1 (0.3)	2 (1.6)	3 (3.4)	0 (0.0)
Cancer	64 (4.1)	40 (4.0)	14 (3.8)	3 (2.4)	7 (7.9)	169 (10.5)
Cardiac or cerebrovascular disease	482 (30.6)	308 (31.1)	102 (27.7)	31 (24.4)	41 (46.1)	714 (44.3)
Chronic kidney failure	113 (7.2)	67 (6.8)	23 (6.2)	9 (7.1)	14 (15.7)	158 (9.8)
Chronic liver disease	52 (3.3)	32 (3.2)	11 (3.0)	2 (1.6)	7 (7.9)	129 (8.0)
Chronic lung disease	125 (7.9)	78 (7.9)	32 (8.7)	5 (3.9)	10 (11.2)	330 (20.5)
Diabetes (type 1 or 2)	411 (26.1)	265 (26.8)	89 (24.2)	30 (23.6)	27 (30.3)	384 (23.8)
Hypertension	689 (43.8)	449 (45.4)	160 (43.5)	42 (33.1)	38 (42.7)	739 (45.8)
Immunocompromised state	163 (10.4)	98 (9.9)	29 (7.9)	17 (13.4)	19 (21.3)	246 (15.3)
Mental health disorder	53 (3.4)	27 (2.7)	13 (3.5)	5 (3.9)	8 (9.0)	99 (6.1)
Neurological disease	35 (2.2)	21 (2.1)	4 (1.1)	3 (2.4)	7 (7.9)	112 (6.9)
Obesity	377 (24.0)	226 (22.9)	120 (32.6)	22 (17.3)	9 (10.1)	147 (9.1)
Charlson comorbidity index score	1.0 [0.0, 2.0]	1.0 [0.0, 2.0]	0.0 [0.0, 2.0]	0.0 [0.0, 1.0]	1.0 [0.0, 3.0]	2.0 [0.0, 3.0]
0	785 (49.9)	476 (48.1)	194 (52.7)	81 (63.8)	34 (38.2)	505 (31.3)
1–2	516 (32.8)	345 (34.9)	114 (31.0)	33 (26.0)	24 (27.0)	516 (32.0)
3–4	172 (10.9)	110 (11.1)	31 (8.4)	10 (7.9)	21 (23.6)	334 (20.7)
≥ 5	100 (6.4)	58 (5.9)	29 (7.9)	3 (2.4)	10 (11.2)	258 (16.0)
COVID-19 vaccination status before hospitalization						
Unvaccinated	1475 (93.8)	989 (100.0)	354 (96.2)	101 (79.5)	31 (34.8)	NA
One dose	18 (1.1)	0 (0.0)	11 (3.0)	5 (3.9)	2 (2.2)	NA
Two doses	57 (3.6)	0 (0.0)	3 (0.8)	19 (15.0)	35 (39.3)	NA
Three doses	23 (1.5)	0 (0.0)	0 (0.0)	2 (1.6)	21 (23.6)	NA
Four doses	0 (0.0)	0 (0.0)	0 (0.0)	0 (0.0)	0 (0.0)	NA
*ICU characteristics*						
Time to ICU admission from COVID-19 symptom onset	10.0 [7.0, 13.0]	10.0 [7.0, 13.0]	10.0 [7.0, 13.0]	10.0 [8.0, 13.0]	3.0 [0.0, 9.0]	NA
Missing	29 (1.8)	12 (1.2)	11 (3.0)	2 (1.6)	4 (4.5)	NA
SAPS 3 score	53.0 [47.0, 60.0]	53.0 [46.0, 60.0]	54.0 [48.0, 60.0]	52.0 [48.0, 57.5]	54.0 [45.0, 61.0]	60.0 [52.0, 68.0]
PaO_2_/FiO_2_ ratio	11.7 [9.2, 15.9]	11.7 [9.3, 15.6]	11.1 [8.9, 15.1]	12.4 [9.4, 16.2]	20.0 [12.2, 44.6]	21.8 [13.1, 33.2]
Missing	193 (12.3)	122 (12.3)	34 (9.2)	11 (8.7)	26 (29.2)	376 (23.3)
Mechanical ventilation	798 (50.7)	553 (55.9)	156 (42.4)	55 (43.3)	34 (38.2)	811 (50.3)
Duration	10.9 [5.3, 22.6]	11.8 [6.3, 23.7]	10.3 [5.1, 24.0]	7.0 [2.9, 14.1]	2.1 [0.5, 4.8]	7.8 [3.1, 16.1]
NIV or HFNO	1074 (68.3)	632 (63.9)	301 (81.8)	101 (79.5)	40 (44.9)	872 (54.1)
Prone positioning	591 (37.6)	415 (42.0)	121 (32.9)	47 (37.0)	8 (9.0)	51 (3.2)
Length of stay in ICU	6.8 [2.7, 16.0]	8.4 [3.3, 18.7]	5.1 [2.3, 11.9]	6.0 [2.4, 12.4]	2.1 [0.8, 4.6]	4.4 [1.7, 12.4]
Length of stay in hospital	24.0 [13.0, 48.0]	27.0 [15.0, 51.0]	23.0 [12.0, 50.0]	21.0 [13.0, 45.0]	13.0 [7.0, 25.0]	21.0 [11.0, 45.0]

Numeric values are presented as median [interquartile range], and categorical values are presented as number (percentage)

*COVID-19* Coronavirus disease 2019, *FiO*_*2*_ Fraction of inspired oxygen, *HFNO* High flow nasal oxygen, *ICU* Intensive care unit, *LRTI* Lower respiratory tract infection, *NA* Not applicable, *NIV* Non-invasive ventilation, *PaO*_*2*_ Partial pressure of oxygen, *SAPS 3* Simplified acute physiology score 3

The 180-day post-discharge mortality was 2% (n = 31) in the All COVID-19 cohort, 1% (n = 14) in the Wild-type cohort, 3% (n = 10) in the Alpha cohort, 3% (n = 4) in the Delta cohort, 3% (n = 3) in the Omicron cohort, and 9% (n = 149) in the LRTI cohort (Table 3). This was 7% (n = 13) in the influenza group and 10% (n = 136) in the other LRTIs group. The point estimates of the adjusted 180-day post-discharge mortality CSHRs were below 0.5 for all COVID-19 cohorts compared with LRTI.

**Table 3:** Long-term outcomes in the cohorts

Outcome	Cohort	Outcome description^a^	Adjusted estimate (95% CI)^b,c^
180-day post-discharge mortality *Cox proportional hazards model regression*	All COVID-19	2.0 (31/1573)	0.26 (0.17–0.38)
	Wild-type	1.4 (14/989)	0.18 (0.10–0.31)
	Alpha	2.7 (10/368)	0.36 (0.19–0.68)
	Delta	3.1 (4/127)	0.47 (0.17–1.27)
	Omicron	3.4 (3/89)	0.39 (0.12–1.21)
	LRTI	9.2 (149/1613)	Reference
180-day hospital readmission *Cox proportional hazards model regression*	All COVID-19	21.0 (331/1573)	0.52 (0.45–0.60)
	Wild-type	20.0 (198/989)	0.46 (0.39–0.55)
	Alpha	21.5 (79/368)	0.53 (0.41–0.68)
	Delta	18.9 (24/127)	0.42 (0.28–0.65)
	Omicron	33.7 (30/89)	0.68 (0.47–0.99)
	LRTI	44.2 (713/1613)	Reference
180-day hospital readmission *Fine-Gray subdistribution hazard model regression*	All COVID-19	21.0 (331/1573)	0.52 (0.45–0.60)
	Wild-type	20.0 (198/989)	0.46 (0.39–0.55)
	Alpha	21.5 (79/368)	0.53 (0.41–0.69)
	Delta	18.9 (24/127)	0.42 (0.27–0.66)
	Omicron	33.7 (30/89)	0.68 (0.48–0.99)
	LRTI	44.2 (713/1613)	Reference
180-day days alive and at home after minus before the critical illness hospitalization *Difference-in-differences analysis*	All COVID-19	− 21 [− 50, − 9]	33.85 (29.70–38.00)
	Wild-type	− 24 [− 54, − 10]	33.54 (29.04–38.03)
	Alpha	− 19 [− 42, − 7]	36.70 (30.60–42.81)
	Delta	− 13 [− 26, − 5]	40.44 (31.25–49.62)
	Omicron	− 24 [− 107, − 8]	16.07 (2.07–30.08)
	LRTI	− 45 [− 171, − 16]	Reference

*CI* Confidence interval, *COVID-19* Coronavirus disease 2019, *LRTI* Lower respiratory tract infection

^a^Percentages and number of individuals are presented for 180-day post-dicharge all-cause mortality and 180-day all-cause hospital readmission. Median and interquartile number of days are presented for 180-day days alive and at home after minus before the critical illness hospitalization

^b^Cause-specific Cox-proportional hazards regression modelling was used to model 180-day post-discharge all-cause mortality. 180-day all-cause hospital readmission was analysed with both Cause-specific Cox-proportional hazards regression modelling and Fine-Gray subdistribution hazard regression modelling. Difference-in-differences analyses was used to model 180-day days alive and at home after minus before the critical illness hospitalization

^c^Models were adjusted for age, sex, region of birth, yearly disposable income quartile, and all studied comorbidities (cancer, cardiac or cerebrovascular disease, chronic kidney failure, chronic liver disease, chronic lung disease, diabetes, hypertension, immunocompromised state, mental health disorder, neurological disease, and obesity). For 180-day post-discharge mortality, adjustments were only made for age and sex due to the low number of events observed

The 180-day hospital readmission was 21% (n = 331) in the All COVID-19 cohort, it was highest in the Omicron cohort, 34% (n = 30), and 44% (n = 713) in the LRTI cohort. This was 39% (n = 73) in the influenza group and 45% (n = 640) in the other LRTIs group. The most common main hospital readmission diagnosis was COVID-19, virus identified in the All COVID-19 cohort (7%, 22/331) and mental and behavioural diseases due to psychoactive substance use (7%, 48/713) in the LRTI cohort (Additional file 1: Fig. S4). When compared with the LRTI cohort, the adjusted CSHR for hospital readmission was 0.52 (0.45–0.60) in the All COVID-19 cohort, 0.46 (0.39–0.55) in the Wild-type cohort, 0.53 (0.41–0.68) in the Alpha cohort, 0.42 (0.28–0.65) in the Delta cohort, and 0.68 (0.47–0.99) in the Omicron cohort. The adjusted SHRs were nearly identical to the adjusted CSHRs.

The median (IQR) number of reduced DAAH after compared with before the critical illness was 21 (9–50) in the All COVID-19 cohort, 24 (10–54) in the Wild-type cohort, 19 (7–42) in the Alpha cohort, 13 (5–26) in the Delta cohort, 24 (8–107) in the Omicron cohort, and 45 (16–171) in the LRTI cohort. This was 28 (11–113) in the influenza group and 48 (16–175) in the other LRTIs group. The All COVID-19 cohort had in adjusted difference-in-difference analyses 34 (95% CI 30–38) more DAAH after compared with before the critical illness episode compared with the LRTI cohort. These numbers were 34 (95% CI 29–38) in the Wild-type cohort, 37 (95% CI 31–43) in the Alpha cohort, 40 (95% CI 31–50) in the Delta cohort, and 16 (95% CI 2–30) in the Omicron cohort.

The most common incident diagnoses were COVID-19 in own medical history in the All COVID-19 cohort (49%, 708/1438), Wild-type COVID-19 cohort (58%, 522/903), Alpha cohort (43%, 141/328), and Delta cohort (31%, 37/120). Malaise and fatigue was the most common diagnosis in the Omicron cohort (12%, 9/75), and dyspnoea was the most common diagnosis in the LRTI cohort (7%, 85/1174) (Additional file 1: Fig. S5).

### 180-day all-cause mortality from the day of ICU admission

The 180-day mortality from the day of ICU admission was 33% (756/2318) in the All COVID-19 cohort, 31% (442/1421) in the Wild-type cohort, 35% (192/551) in the Alpha cohort, 35% (67/190) in the Delta cohort, 35% (55/156) in the Omicron cohort, and 34% (768/2272) in the LRTI cohort. This was 27% (65/244) in the influenza group and 35% (703/2028) in the other LRTIs group. The overall and age-stratified cumulative incidences are presented in Additional file 1: Fig. S6. Overall, among individuals who died within 180 days, more than 90% had died within the first 90 days since the date of the ICU admission.

## Discussion

In this population-based study including 2385 COVID-19 and 2380 LRTI patients from all ICUs in Stockholm County, we found all variant cohorts (Wild-type, Alpha, Delta, Omicron) to have an increased hazard of in-hospital mortality compared with the LRTI cohort. However, among those patients discharged alive from the hospital, more favourable outcomes in the COVID-19 cohort compared with the LRTI cohort were observed. This included lower 180-day post-discharge all-cause mortality and hospital re-admission as well as more DAAH.

To our knowledge, this is the first study to examine the trajectory of patients in the ICU with COVID-19 and different SARS-CoV-2 variants compared with other LRTI patients in a population-based setting. Studies are often limited by lack of data from both before, during, and after the critical illness episode, in particular data on primary care and other outpatient care services. A French study including more than 100,000 critically ill COVID-19 patients and around 19,000 pre-pandemic influenza patients found a 25% hospital mortality in COVID-19 patients and a 21% hospital mortality in influenza patients, with an increased adjusted mortality hazard ratio in the COVID-19 cohort [23]. However, this study was conducted during a pre-vaccination era up until June 2021. Regarding long-term outcomes, we observed a rather low 180-day post-discharge mortality (2%) in the COVID-19 cohort. This finding is similar to a study including patients admitted to 60 Spanish ICUs during the first wave of the pandemic, where a one-year mortality rate from day of ICU admission was 35%, with 2% dying after hospital discharge [2]. In our study, not only 180-day post-discharge mortality, but also the hospital readmission and DAAH outcomes were more favourable in the COVID-19 cohort compared with the LRTI cohort. These findings should be contrasted to those observed in an Australian observational study investigating new disability at 6 months in mechanically ventilated COVID-19 versus non COVID-19 patients, where no difference was observed [10]. Important differences between our study and the Australian study should be noticed. First, the Australian study restricted the study population to patients mechanically ventilated for 24 h or more, whereas we included all critically ill patients. Furthermore, patients were actively followed, whereas we considered data from health registries. Both approaches have their inherent strengths and limitations. We might not have accurately captured the health status of patients by relying on data from health registries, whereas in the Australian study, recall bias might have been introduced when patients should assess baseline function 6 months later. Furthermore, our study compared COVID-19 patients with other LRTIs, whereas Hodgson et al. included all non-COVID-19 patients.

Our findings of rather similar cumulative incidences and SHRs for in-hospital mortality across the different SARS-CoV-2 variant cohorts are in line with two French studies, observing no difference in in-ICU mortality or 28-day mortality between the Delta and the Omicron period [7, 8]. Importantly, the actual number of patients included during each variant period have decreased (1421 Wild-type, 551 Alpha, 190 Delta, and 223 Omicron), likely caused by increased population-level immunity from vaccinations and previous infections and less severe intrinsic of in particular Omicron compared with previous variants. It is also evident that patients in the Omicron cohort were older and had more comorbidities compared with the other variant cohorts, factors greatly influencing the risk of severe COVID-19 disease outcomes.

Strengths of our study include the multicentre setting including critically ill patients from all ICUs in Stockholm County, linking several population-based data sources with high coverage. This enabled us to characterize and account for differences in patient characteristics, including underlying medical conditions and sociodemographic factors. Furthermore, we were able to investigate DAAH, a patient-centred outcome considering death, hospitalization, outpatient care services, nursing home, home care services, and telecare services [16, 24]. Finally, by including not only COVID-19 patients from the earlier phases of the pandemic, we were able to assess COVID-19 critical illness throughout different variant periods, demonstrating how the Omicron cohort became more similar to the LRTI cohort. Regarding limitations, we could only include a fairly low number of omicron patients. Although we restricted our analyses to patients with a COVID-19 diagnostic code we cannot fully ascertain whether patients were admitted due to or with their respiratory infection. Yet, if there was non-differential misclassification between the different variants and LRTI cohorts it would result in limited bias on the relative estimates. Furthermore, although describing and accounting for medical conditions, it is highly plausible that administrative codes don’t give a granular enough picture of the severity of these conditions, possibly resulting in residual confounding. The effect of COVID-19 vaccination was not analyzed in this study given the low number of patients having received two doses or more before the hospitalization (n = 196). It is plausible that COVID-19 vaccination status before the hospitalization will have an effect of the risk of severe outcomes, even among patients requiring intensive care treatment. Finally, ICU capacities and admission criteria differ substantially by geographical setting, thus making it more difficult to evaluate the generalizability of our study and similar studies. The number of critical care beds per 100,000 inhabitants were shown to vary substantially across Europe in a study from 201, with 5.8 beds in Sweden compared with for example 29.2 beds in Germany, 6.6 beds in United Kingdom, and around 12 beds in both Italy and France [25].

## Conclusion

In large multicentre cohorts of critically ill COVID-19 and LRTI patients, we found that all SARS-CoV-2 variant periods had an increased hazard of in-hospital mortality compared with the LRTI cohort. However, among those discharged alive from the hospital, more favourable post-discharge outcomes were observed in the COVID-19 cohorts, indicating different clinical trajectories and patient populations in COVID-19 critical illness compared with other LRTIs.

### Supplementary Information

**Additional file 1:** Supplementary material for Clinical outcomes during and beyond different COVID-19 critical illness variant periods compared with other lower respiratory tract infections.

## Data Availability

The individual participant data underlying this article were subject to ethical approval and cannot be shared publicly. Data from the deidentified administrative health registry are not freely available due to protection of the personal integrity of the participants.
