# Supplementary material for: Clinical outcomes during and beyond different COVID-19 critical illness variant periods compared with other lower respiratory tract infections
Source: Crit Care. 2023 Nov 6;27:427. doi: 10.1186/s13054-023-04722-0 (PMC10629059; doi:10.1186/s13054-023-04722-0)
Supplement: Supplementary file 1 — Additional file 1: Supplementary material for Clinical outcomes during and beyond different COVID-19 critical illness variant periods compared with other lower respiratory tract infections. [file 13054_2023_4722_MOESM1_ESM.docx]

Supplementary material for

**Clinical outcomes during and beyond different COVID-19 critical illness variant periods compared with other lower respiratory tract infections**

Pontus Hedberg, Nicholas Baltzer, Fredrik Granath, Michael Fored, Johan Mårtensson, Pontus Nauclér

Correspondence to [pontus.hedberg@ki.se](mailto:pontus.hedberg@ki.se)

**Table of contents**

| **Content** | **Page** |
| --- | --- |
| Text S1. Descriptions of data sources | 3 |
| Table S1. Definitions of study outcomes and other collected variables | 4 to 9 |
| Figure S1. Number of individuals admitted to the ICU per calendar month in the cohorts | 10 |
| Table S2. Characteristics of Delta and Omicron patients by vaccination status before hospitalization | 11-12 |
| Table S3. Characteristics of the influenza and other LRTIs groups | 13-14 |
| Figure S2. Overall and age-stratified cumulative incidences of in-hospital mortality and alive hospital discharge in each cohort | 15 |
| Table S4. Characteristics of the influenza and other LRTIs groups discharged alive from the hospital | 16-17 |
| Figure S3. Age-stratified in-hospital mortality rates in the cohorts | 18 |
| Figure S4. Most common main diagnoses during the hospital readmission in the cohorts | 19 |
| Figure S5. Most common incident diagnoses registered by physicians after hospital discharge in the cohorts | 20 |
| Figure S6. Overall and age-stratified cumulative incidences of 180-day mortality from day of ICU admission in each cohort | 21 |
| Supplemental references | 22 |

**Text S1. Descriptions of data sources**

**Swedish Intensive Care Registry (SIR):** SIR is a national quality register for intensive care which was established in 2001.[1] SIR prospectively collects data from intensive care unit admission in Sweden, currently including data from all 83 intensive care units in Sweden.[2] Data on all COVID-19 related admissions to an intensive care unit for the study population were used in this study.

**Stockholm regional healthcare data warehouse (VAL):** The Stockholm Regional Council operates the healthcare data warehouse VAL, which contains data from multiple administrative healthcare databases.[3] This data includes information on inpatient stays, outpatient specialist visits and primary care visits (coverage of around 94%) reimbursed by Region Stockholm.[4] Furthermore, the data warehouse contains information on demographics, migration status, nursing home residency, home care services, and collected drug prescriptions. Data on all such characteristics for the study population were used in this study.

**SmiNet:** SmiNet is the electronic system used for surveillance of communicable diseases in Sweden, owned and operated by PHAS and the communicable disease control units in Sweden.[5] It is used for surveillance of more than 60 notifiable diseases, including COVID-19, according to the Communicable Diseases Act and the Communicable Diseases Ordinance.[6, 7] Data on all polymerase chain reaction (PCR) tests positive for severe acute respiratory syndrome coronavirus 2 (SARS-CoV-2) for the study population were used in this study.

**National Vaccination Register (NVR):** All vaccinations within national vaccination programs and vaccinations against COVID-19 should according to Swedish law be reported to the NVR, which is governed by the Public Health Agency of Sweden (PHAS).[8] Data on administered COVID-19 vaccine doses for the study population were used in this study.

**Statistics Sweden:** Statistics Sweden is a governmental agency supplying statistics for decision making, debate, and research.[9] This includes several registers such as the Total Population Register (TPR), and the Integrated Database for Labour Market Research (LISA). Data on region of birth and disposable family income for the study population were used in this study.

**Table S1. Definitions of study outcomes and other collected variables**

| **Variable** | **Data sources** | **Missing data** | **Definition** | **Time period** | **Possible values** |
| --- | --- | --- | --- | --- | --- |
| **Study outcomes** |  |  |  |  |  |
| In-hospital all-cause mortality | VAL | No | Date of death (of any cause) any time from day of admission to the ICU to day of hospital discharge | Day of admission to the ICU to day of hospital discharge | Yes, No |
| 180-day post-discharge all-cause mortality | VAL | No | Date of death (of any cause) any time from day after hospital discharge and 180 days onwards | 0 to 180 days from day after hospital discharge | Yes, No |
| 180-day all-cause hospital readmission | VAL | No | Date of hospital readmission (of any cause) any time from day after hospital discharge and 180 days onwards | 0 to 180 days from day after hospital discharge | Yes, No |
| Days alive and at home after compared with before the critical illness | VAL | No | Number of days alive and free from hospitalization, outpatient care, nursing home, home care services, and telephone calls to the Swedish National Medical Advisory Service (called 1177in Swedish) from 1 day to 180 days after discharge from the ICU-associated hospital admission  Minus  Number of days alive and free from hospitalization, outpatient care, nursing home, home care services, and telephone calls to the Swedish National Medical Advisory Service (called 1177in Swedish) from from 181 days to 1 day before admission to the ICU-associated hospitalization | 1 day to 180 days from day after discharge from the ICU-associated hospital admission  181 days to 1 day before admission to the ICU-associated hospitalization | -180 to 180 days |
| Incident diagnoses registered by physicians during follow-up | VAL | No | The ten most common ICD-10 diagnosis codes registered by physicians during follow-up, which the individual did not have registered any time from three years before hospital admission or during the hospitalization | 1 day to 180 days from day after discharge from the ICU-associated hospital admission | Ten most common diagnoses for all cohorts |
| **Other collected variables** | | | | | |
| SARS-CoV-2 variant | SmiNet | No | **Wild-type:** Positive test any time from 31 January 2020 to 14 February 2021 or whole genome sequence classified as Wild-type  **Alpha:** Positive test any time from 15 February 2021 to 27 June 2021 or whole genome sequence classified as Alpha  **Delta:** Positive test any time from 28 June 2021 to 26 December 2021 or whole genome sequence classified as Delta  **Omicron:** Positive test any time from 27 December 2021 to 15 September 2022 or whole genome sequence classified as Omicron | Positive SARS-CoV-2 test | Wild-type, Alpha, Delta, Omicron |
| Sex | VAL | No | Sex of individual | Birth | Male, Female |
| Age | VAL | No | Age the year of hospital admission | Year of hospital admission | 18 to 99 years |
| Age category | VAL | No | Age category the year of hospital admission | Year of hospital admission | 18 to 44 years, 44 to 64 years, 65 years or older |
| Region of birth | Statistics Sweden | Yes, for 4 individuals | According to the United Nations geoscheme | Birth | Africa, The Americas, Asia or Oceania, Europe, Sweden, Missing |
| Yearly disposable income quartile | Statistics Sweden | Yes, for 9 individuals | The birthyear stratified yearly disposable income quartile the year before the year of hospital admission | Year of hospital admission | Quartile 1, Quartile 2, Quartile 3, Quartile 4, Missing |
| Cancer | VAL | No | **ICD-10:** All codes from C00.X to C97.X (besides C44.X), Z51.0, Z51.1  **KVÅ:** DT107, DT108, DT112, DT116, DT135, DV070, DV071, DV134 | One day to one year before hospital admission  One day to one year before hospital admission | Yes, No |
| Cardiac or cerebrovascular disease | VAL | No | **ICD-10:** I20.X, I21.X, I22.X, I23.X, I24.X, I25.X, I26.X, I27.X, I42.X, I48.X, I50.X, I61.X, I63.X, I64.X | One day to five years before hospital admission | Yes, No |
| Chronic kidney failure | VAL | No | **ICD-10:** N18.X  **ICD-10:** Z49.1, Z49.2 (should be registered at least twelve times during the time period)  **ICD-10:** Z99.2  **KVÅ:** DR016, DR024 (should be registered at least twelve times during the time period) | One day to five years before hospital admission One day to one year before hospital admission  One day to one year before hospital admission  One day to one year before hospital admission | Yes, No |
| Chronic liver disease | VAL | No | **ICD-10:** B18.X, K70.X, K71.7, K72.X, K74.6, K75.X | One day to five years before hospital admission | Yes, No |
| Chronic lung disease | VAL | No | **ICD-10:** D86.0, D86.2, E84.X, J43.X, J44.X, J47.X, J70.3, J84.X, J98.2 | One day to five years before hospital admission | Yes, No |
| Diabetes (type 1 or 2) | VAL | No | **ICD-10:** E10.X, E11.X | One day to five years before hospital admission | Yes, No |
| Hypertension | VAL | No | **ICD-10:** I10.X, I11.X, I12.X, I13.X, I14.X, I15.X (should be registered at least twice during the time period) | One day to five years before hospital admission | Yes, No |
| Immunocompromised state | VAL | No | **ATC:** H02AB.X (should be registered at least twice during the time period)  **ATC:** L01.X  **ATC:** L04.X  **ICD-10:** B20.X, B21.X, B22.X, B23.X, B24.X, D57.0, D57.1, D80.X, D81.X, D82.X, D83.X, D84.X, Z94.0, Z94.1, Z94.2, Z94.3, Z94.4, Z94.8  **KVÅ:** DR04.1, DR04.2, DR04.4, DR04.6, DR04.7  **KVÅ:** H02AB.X (should be registered at least twice during the time period)  **KVÅ:** L01.X  **KVÅ:** L04.X | One day to half a year before hospital admission    One day to one year before hospital admission One day to half a year before hospital admission One day or more before hospital admission  One day to three years before hospital admission  One day to half a year before hospital admission One day to one year before hospital admission  One day to half a year before hospital admission | Yes, No |
| Mental health disorder | VAL | No | **ICD-10:** F20.X-F29.X, F31.X | One day to five years before hospital admission | Yes, No |
| Neurologic disease | VAL | No | **ICD-10:** F00.X, F01.X, F02.X, F03.X, G10.X, G12.2, G20.X, G30.X, G35.X, G70.X, G71.X, G80.X | One day to five years before hospital admission | Yes, No |
| Obesity | VAL | No | **ICD-10:** E66.X | One day to five years before hospital admission | Yes, No |
| Charlson Comorbidity Index score | VAL | No | Based on all diagnosis code included in the Charlson Comorbidity Index | One day to five years before hospital admission | 0 to 18 |
| COVID-19 vaccination status before hospitalization | NVR | No | Number of COVID-19 vaccine doses received any time up until 14 days before hospital admission | 27 December 2020 up until 14 days before hospital admission | Unvaccinated, 1 dose, 2 doses, 3 doses, 4 doses |
| Time to ICU admission from COVID-19 symptom onset | SIR | Yes, for 56 individuals | Time since symptom onset to ICU-admission | - | -6 to 127 days, Missing |
| SAPS 3 score | SIR | No | SAPS 3 score at ICU-admission | 1 hour before to 1 hour after ICU admission | 24 to 125 points |
| PaO_2_/FiO_2_ ratio | SIR | Yes, for 782 individuals | PaO_2_/FiO_2_ ratio at ICU-admission | 1 hour before to 1 hour after ICU admission | 3.2 to 302.0, Missing |
| Mechanical ventilation | SIR | No | **KVÅ:** DG021 | First ICU admission to last ICU discharge | Yes, No |
| Duration of mechanical ventilation | SIR | No | Duration of mechanical ventilation during the ICU-stay | First intubation to last extubation | 0 to 138 days |
| NIV or HFNO | SIR | No | **KVÅ:** DG023, DG028 | First ICU admission to last ICU discharge | Yes, No |
| Prone positioning | SIR | No | **KVÅ:** SR320 | First ICU admission to last ICU discharge | Yes, No |
| Length of stay in ICU | SIR | No | Number of days in ICU during the hospitalization | First ICU admission to last ICU discharge | 0 to 161 days |
| Length of stay in hospital | VAL | Yes, for 10 individuals | Number of days from hospital admission to hospital discharge | Hospital admission to hospital discharge | 0 to 514 days, Missing |

**Abbreviations:** ATC=Anatomical Therapeutic Chemical, COVID-19=Coronavirus disease 2019, ICD-10=International Statistical Classification of Diseases and Related Health Problems 10^th^ Revision; ICU=Intensive care unit, FiO_2_=Fraction of inspired oxygen, HFNO=High Flow Nasal Oxygen, KVÅ=Klassifikation av vårdåtgärder (Swedish for classification of healthcare procedures), NIV=Non-invasive ventilation, NVR=National Vaccination Register, PaO_2_=Partial pressure of oxygen, SAPS 3=Simplified Acute Physiology Score 3, SARS-CoV-2=Severe acute respiratory syndrome coronavirus 2, SIR=Swedish Intensive Care Registry, VAL=Stockholm regional healthcare data warehouse

**Figure S1. Number of individuals admitted to the ICU per calendar month in the cohorts**

**Abbreviations:** ICU=Intensive care unit, LRTI=Lower respiratory tract infection

**Table S2. Characteristics of Delta and Omicron patients by vaccination status before hospitalization**

| **Variable** | **Unvaccinated or 1 dose (n=221)** | **>2 doses (n=192)** |
| --- | --- | --- |
| **Baseline characteristics** |  |  |
| Delta period | 145 (65.6) | 45 (23.4) |
| Omicron period | 76 (34.4) | 147 (76.6) |
| Male sex | 150 (67.9) | 124 (64.6) |
| Age, years | 58.0 [48.0, 70.0] | 70.0 [59.0, 77.2] |
| 18-44 | 43 (19.5) | 23 (12.0) |
| 45-64 | 99 (44.8) | 51 (26.6) |
| 65 or older | 79 (35.7) | 118 (61.5) |
| Region of birth |  |  |
| Africa | 16 (7.2) | 10 (5.2) |
| The Americas | 9 (4.1) | 3 (1.6) |
| Asia or Oceania | 63 (28.5) | 18 (9.4) |
| Europe | 45 (20.4) | 26 (13.5) |
| Sweden | 86 (38.9) | 135 (70.3) |
| Missing | 2 (0.9) | 0 (0.0) |
| Yearly disposable income quartile |  |  |
| Quartile 1 | 129 (58.4) | 70 (36.5) |
| Quartile 2 | 42 (19.0) | 59 (30.7) |
| Quartile 3 | 31 (14.0) | 39 (20.3) |
| Quartile 4 | 14 (6.3) | 23 (12.0) |
| Missing | 5 (2.3) | 1 (0.5) |
| Cancer | 7 (3.2) | 21 (10.9) |
| Cardiac or cerebrovascular disease | 40 (18.1) | 73 (38.0) |
| Chronic kidney failure | 15 (6.8) | 37 (19.3) |
| Chronic liver disease | 4 (1.8) | 8 (4.2) |
| Chronic lung disease | 12 (5.4) | 26 (13.5) |
| Diabetes (type 1 or 2) | 54 (24.4) | 57 (29.7) |
| Hypertension | 74 (33.5) | 105 (54.7) |
| Immunocompromised state | 19 (8.6) | 55 (28.6) |
| Mental health disorder | 10 (4.5) | 9 (4.7) |
| Neurological disease | 5 (2.3) | 14 (7.3) |
| Obesity | 30 (13.6) | 25 (13.0) |
| Charlson comorbidity index score | 0.0 [0.0, 1.0] | 2.0 [0.0, 3.0] |
| 0 | 148 (67.0) | 63 (32.8) |
| 1-2 | 47 (21.3) | 63 (32.8) |
| 3-4 | 18 (8.1) | 37 (19.3) |
| >5 | 8 (3.6) | 29 (15.1) |
| COVID-19 vaccination status before hospitalization |  |  |
| Unvaccinated | 210 (95.0) | 0 (0.0) |
| One dose | 11 (5.0) | 0 (0.0) |
| Two doses | 0 (0.0) | 102 (53.1) |
| Three doses | 0 (0.0) | 67 (34.9) |
| Four doses | 0 (0.0) | 23 (12.0) |
| **ICU characteristics** |  |  |
| Time to ICU admission from COVID-19 symptom onset | 10.0 [7.0, 14.0] | 4.0 [0.0, 8.0] |
| Missing | 7 (3.2) | 11 (5.7) |
| SAPS 3 score | 55.0 [48.0, 63.0] | 61.0 [53.0, 73.0] |
| PaO_2_/FiO_2_ ratio | 11.7 [8.7, 15.6] | 18.2 [11.1, 34.7] |
| Missing | 26 (11.8) | 36 (18.9) |
| Mechanical ventilation | 118 (53.4) | 96 (50.0) |
| Duration | 8.2 [3.5, 14.7] | 3.7 [0.8, 9.7] |
| NIV or HFNO | 162 (73.3) | 100 (52.1) |
| Prone positioning | 79 (35.7) | 34 (17.7) |
| Length of stay in ICU | 7.0 [2.3, 14.2] | 3.1 [1.0, 7.7] |
| Length of stay in hospital | 18.0 [11.0, 31.0] | 14.0 [7.0, 28.8] |
| Missing | 0 (0.0) | 2 (1.0) |
| In-hospital mortality | 67 (30.3) | 73 (38.0) |

**Note:** Numeric values are presented as median [interquartile range], and categorical values are presented as number (percentage).

**Abbreviations:** COVID-19=Coronavirus disease 2019, FiO_2_=Fraction of inspired oxygen, HFNO=High Flow Nasal Oxygen, ICU=Intensive care unit, LRTI=Lower respiratory tract infection, NIV=Non-invasive ventilation, PaO_2_=Partial pressure of oxygen, SAPS 3=Simplified Acute Physiology Score 3

**Table S3. Characteristics of the influenza and other LRTIs groups**

| **Variable** | **Influenza (n=246)** | **Other LRTIs (n=2,134)** |
| --- | --- | --- |
| **Baseline characteristics** |  |  |
| Male sex | 144 (58.5) | 1,362 (63.8) |
| Age, years | 63.5 [51.0, 74.0] | 67.0 [56.0, 75.0] |
| 18-44 | 38 (15.4) | 262 (12.3) |
| 45-64 | 92 (37.4) | 642 (30.1) |
| 65 or older | 116 (47.2) | 1,230 (57.6) |
| Region of birth |  |  |
| Africa | 12 (4.9) | 59 (2.8) |
| The Americas | 6 (2.4) | 21 (1.0) |
| Asia or Oceania | 32 (13.0) | 146 (6.8) |
| Europe | 36 (14.6) | 266 (12.5) |
| Sweden | 160 (65.0) | 1,641 (76.9) |
| Missing | 0 (0.0) | 1 (0.0) |
| Yearly disposable income quartile |  |  |
| Quartile 1 | 115 (46.7) | 892 (41.8) |
| Quartile 2 | 60 (24.4) | 528 (24.7) |
| Quartile 3 | 40 (16.3) | 383 (17.9) |
| Quartile 4 | 31 (12.6) | 329 (15.4) |
| Missing | 0 (0.0) | 2 (0.1) |
| Cancer | 27 (11.0) | 300 (14.1) |
| Cardiac or cerebrovascular disease | 80 (32.5) | 690 (32.3) |
| Chronic kidney failure | 23 (9.3) | 189 (8.9) |
| Chronic liver disease | 15 (6.1) | 145 (6.8) |
| Chronic lung disease | 41 (16.7) | 410 (19.2) |
| Diabetes (type 1 or 2) | 61 (24.8) | 479 (22.4) |
| Hypertension | 101 (41.1) | 1,014 (47.5) |
| Immunocompromised state | 57 (23.2) | 380 (17.8) |
| Mental health disorder | 10 (4.1) | 128 (6.0) |
| Neurological disease | 18 (7.3) | 158 (7.4) |
| Obesity | 18 (7.3) | 165 (7.7) |
| Charlson comorbidity index score | 1.0 [0.0, 3.0] | 1.0 [0.0, 3.0] |
| 0 | 96 (39.0) | 772 (36.2) |
| 1-2 | 69 (28.0) | 639 (29.9) |
| 3-4 | 49 (19.9) | 383 (17.9) |
| >5 | 32 (13.0) | 340 (15.9) |
| **ICU characteristics** |  |  |
| SAPS 3 score | 62.0 [54.0, 72.0] | 63.0 [54.0, 73.0] |
| PaO_2_/FiO_2_ ratio | 21.2 [13.4, 30.0] | 19.4 [12.0, 30.8] |
| Missing | 44 (17.9) | 443 (20.8) |
| Mechanical ventilation | 143 (58.1) | 1164 (54.5) |
| Duration | 7.2 [2.7, 16.4] | 7.2 [3.1, 15.2] |
| NIV or HFNO | 151 (61.4) | 1,181 (55.3) |
| Prone positioning | 16 (6.5) | 88 (4.1) |
| Length of stay in ICU | 4.0 [1.6, 13.6] | 4.8 [1.8, 12.8] |
| Length of stay in hospital | 17.0 [10.0, 32.8] | 19.0 [9.0, 40.0] |
| Missing | 0 (0.0) | 7 (0.3) |

**Note:** Numeric values are presented as median [interquartile range], and categorical values are presented as number (percentage).

**Abbreviations:** FiO_2_=Fraction of inspired oxygen, HFNO=High Flow Nasal Oxygen, ICU=Intensive care unit, LRTI=Lower respiratory tract infection NIV=Non-invasive ventilation, PaO_2_=Partial pressure of oxygen, SAPS 3=Simplified Acute Physiology Score 3

**Figure S2. Overall and age-stratified cumulative incidences of in-hospital mortality and alive hospital discharge in each cohort**

**Note:** Cumulative incidence (percentage) of 120-day in-hospital all-cause mortality and alive hospital discharge in the all COVID-19, Wild-type, Alpha, Delta, Omicron, and LRTI cohort.

**Abbreviations:** COVID-19=Coronavirus disease 2019, LRTI=Lower respiratory tract infection

**Table S4. Characteristics of the influenza and other LRTIs groups discharged alive from the hospital**

| **Variable** | **Influenza (n=189)** | **Other LRTIs (n=1,424)** |
| --- | --- | --- |
| **Baseline characteristics** |  |  |
| Male sex | 104 (55.0) | 904 (63.5) |
| Age, years | 61.0 [48.0, 73.0] | 65.0 [53.0, 73.0] |
| 18-44 | 35 (18.5) | 228 (16.0) |
| 45-64 | 78 (41.3) | 478 (33.6) |
| 65 or older | 76 (40.2) | 718 (50.4) |
| Region of birth |  |  |
| Africa | 12 (6.3) | 41 (2.9) |
| The Americas | 4 (2.1) | 12 (0.8) |
| Asia or Oceania | 28 (14.8) | 110 (7.7) |
| Europe | 26 (13.8) | 177 (12.4) |
| Sweden | 119 (63.0) | 1,084 (76.1) |
| Yearly disposable income quartile |  |  |
| Quartile 1 | 96 (50.8) | 616 (43.3) |
| Quartile 2 | 42 (22.2) | 351 (24.6) |
| Quartile 3 | 30 (15.9) | 250 (17.6) |
| Quartile 4 | 21 (11.1) | 207 (14.5) |
| Cancer | 20 (10.6) | 149 (10.5) |
| Cardiac or cerebrovascular disease | 67 (35.4) | 647 (45.4) |
| Chronic kidney failure | 18 (9.5) | 140 (9.8) |
| Chronic liver disease | 14 (7.4) | 115 (8.1) |
| Chronic lung disease | 36 (19.0) | 294 (20.6) |
| Diabetes (type 1 or 2) | 48 (25.4) | 336 (23.6) |
| Hypertension | 85 (45.0) | 654 (45.9) |
| Immunocompromised state | 40 (21.2) | 206 (14.5) |
| Mental health disorder | 7 (3.7) | 92 (6.5) |
| Neurological disease | 13 (6.9) | 99 (7.0) |
| Obesity | 18 (9.5) | 129 (9.1) |
| Charlson comorbidity index score | 1.0 [0.0, 3.0] | 2.0 [0.0, 4.0] |
| 0 | 69 (36.5) | 436 (30.6) |
| 1-2 | 53 (28.0) | 463 (32.5) |
| 3-4 | 40 (21.2) | 294 (20.6) |
| >5 | 27 (14.3) | 231 (16.2) |
| **ICU characteristics** |  |  |
| SAPS 3 score | 60.0 [52.0, 69.0] | 59.0 [51.0, 68.0] |
| PaO_2_/FiO_2_ ratio | 22.4 [13.5, 30.0] | 22.0 [13.0, 33.8] |
| Missing | 38 (20.1) | 335 (23.5) |
| Mechanical ventilation | 100 (52.9) | 711 (49.9) |
| Duration | 8.0 [2.9, 15.7] | 7.8 [3.1, 16.2] |
| NIV or HFNO | 116 (61.4) | 756 (53.1) |
| Prone positioning | 11 (5.8) | 40 (2.8) |
| Length of stay in ICU | 3.8 [1.5, 12.5] | 4.5 [1.7, 12.3] |
| Length of stay in hospital | 18.0 [11.0, 36.0] | 22.0 [10.8, 47.0] |

**Note:** Numeric values are presented as median [interquartile range], and categorical values are presented as number (percentage).

**Abbreviations:** FiO_2_=Fraction of inspired oxygen, HFNO=High Flow Nasal Oxygen, ICU=Intensive care unit, LRTI=Lower respiratory tract infection, NIV=Non-invasive ventilation, PaO_2_=Partial pressure of oxygen, SAPS 3=Simplified Acute Physiology Score 3

**Figure S3. Age-stratified in-hospital mortality rates in the cohorts**

**Note:** The numbers in each cell represent the percentage dying in the hospital (number of individuals dying in the hospital / number of individuals in each cell).

**Abbreviations:** COVID-19=Coronavirus disease 2019, LRTI=Lower respiratory tract infection

**Figure S4. Most common main diagnoses during the hospital readmission in the cohorts**


**Note:** The numbers in each cell represent the percentage of all hospital readmitted individuals with a diagnosis (number of hospital readmitted individuals with a diagnosis / number of hospital readmitted individuals). Blank cells mean no individual was diagnosed with that condition.

**Abbreviations:** COVID-19=Coronavirus disease 2019, ICD-10=International Statistical Classification of Diseases and Related Health Problems 10^th^ Revision

**Figure S5. Most common incident diagnoses registered by physicians after hospital discharge in the cohorts**

**Note:** The 10 most commonly registered incident ICD-10 codes (from physicians only) during follow-up were identified for each cohort. The percentage being diagnosed with these diagnoses were then calculated for each cohort. Individuals who had the diagnosis registered before the start of the long-term follow-up were excluded from the analyses. The numbers in each cell represent the percentage diagnosed with the specified code (number of individuals with the specified code / number of individuals in each cell). Blank cells mean no individual was diagnosed with that condition.

**Abbreviations:** ICD-10=International Statistical Classification of Diseases and Related Health Problems 10th Revision

**Figure S6. Overall and age-stratified cumulative incidences of 180-day mortality from day of ICU admission in each cohort**

**Note:** Cumulative incidence (percentage) of 120-day in-hospital all-cause mortality and alive hospital discharge in the all COVID-19, Wild-type, Alpha, Delta, Omicron, and LRTI cohort.

**Abbreviations:** COVID-19=Coronavirus disease 2019, LRTI=Lower respiratory tract infection

**Supplemental references**

1. Mårtensson J, Engerström L, Walther S, et al (2020) COVID-19 critical illness in Sweden: characteristics and outcomes at a national population level. Crit Care Resusc 22:312–320. https://doi.org/10.51893/2020.4.OA3

2. Zettersten E, Engerström L, Bell M, et al (2021) Long-term outcome after intensive care for COVID-19: differences between men and women—a nationwide cohort study. Crit Care 25:86. https://doi.org/10.1186/s13054-021-03511-x

3. Hergens M-P, Bell M, Haglund P, et al (2022) Risk factors for COVID-19-related death, hospitalization and intensive care: a population-wide study of all inhabitants in Stockholm. Eur J Epidemiol 37:157–165. https://doi.org/10.1007/s10654-021-00840-7

4. Hedberg P, Granath F, Bruchfeld J, et al (2023) Post COVID‐19 condition diagnosis: A population‐based cohort study of occurrence, associated factors, and healthcare use by severity of acute infection. J Intern Med 293:246–258. https://doi.org/10.1111/joim.13584

5. Rolfhamre P, Janson A, Arneborn M, Ekdahl K (2006) SmiNet-2: Description of an internet-based surveillance system for communicable diseases in Sweden. Euro Surveill Bull Eur Sur Mal Transm Eur Commun Dis Bull 11:15—16. https://doi.org/10.2807/esm.11.05.00626-en

6. Notifiable diseases - The Public Health Agency of Sweden. https://www.folkhalsomyndigheten.se/the-public-health-agency-of-sweden/communicable-disease-control/surveillance-of-communicable-diseases/notifiable-diseases/. Accessed 9 Mar 2023

7. Swedish Government Smittskyddslag (2004:168). https://rkrattsbaser.gov.se/sfst?bet=2004:168. Accessed 29 Sep 2022

8. Public Health Agency of Sweden Nationella vaccinationsregistret — Folkhälsomyndigheten. https://www.folkhalsomyndigheten.se/smittskydd-beredskap/vaccinationer/nationella-vaccinationsregistret/. Accessed 9 Mar 2023

9. Statistics Sweden About Statistics Sweden. In: Stat. Cent. https://www.scb.se/en/About-us/. Accessed 29 Sep 2022
